# Co-designing interventions with multiple stakeholders to address barriers and promote equitable access to HIV Pre-Exposure Prophylaxis (PrEP) in Black women in England

**DOI:** 10.1186/s12889-025-23023-5

**Published:** 2025-05-17

**Authors:** Flavien Coukan, Wezi Thamm, Fola Afolabi, Keitumetse-Kabelo Murray, Adam Pattison Rathbone, John Saunders, Christina Atchison, Helen Ward

**Affiliations:** 1 National Institute for Health Research Applied Research Collaboration North West London, Chelsea and Westminster Hospital, London, UK; 2 Patient Experience Research Centre, School of Public Health, Imperial College London, White City Campus, 90 Wood Lane, London, W12 7TA UK; 3 School of Pharmacy, Newcastle University, Newcastle Upon Tyne, UK; 4 The Sophia Forum, London, UK; 5 Hillingdon AIDS Response Trust (HART), London, UK; 6 Youth Involvement and Engagement Lab, London, UK; 7 Blood Safety, Hepatitis, Sexually Transmitted Infections (STI) and HIV Division, UK Health Security Agency, London, UK; 8 National Institute for Health Research Imperial Biomedical Research Centre, London, UK

**Keywords:** HIV Pre-exposure Prophylaxis (PrEP), Sexual health, Community-based participatory research, Health services accessibility, Quality improvement, Health equity, Stakeholder participation

## Abstract

**Background:**

Black women are among the populations most underserved by HIV pre-exposure prophylaxis (PrEP) in England, despite higher risk of HIV acquisition. Previous research mostly focused on men who have sex with men (MSM), often neglecting Black women, and overfocused on patient-level barriers while overlooking provider and system-level factors. This study addresses these gaps by investigating barriers and facilitators to PrEP access by involving multiple stakeholders and exploring co-design strategies to tackle these barriers.

**Methods:**

The study used a structured two-phased qualitative approach. In Phase 1, focus groups (FG) were undertaken across three stakeholder streams: Black women, healthcare professionals (HCPs), and a group combining Black women and HCPs. FG allowed for consensus-building exercises on key barriers and facilitators to PrEP access, and their transcripts were analysed via thematic framework analysis using the Capability, Opportunity, Motivation and Behaviour model of behaviour change. In Phase 2, co-design workshops were conducted with the same stakeholder groups to develop interventions targeting the barrier identified as most important using the Behaviour Change Wheel framework. Interventions were evaluated against the APEASE criteria.

**Results:**

Phase 1 identified six key barriers: HIV/PrEP knowledge gaps, restrictive policies, cultural stigma, healthcare system distrust, gendered relationship dynamics, and suboptimal PrEP use. Six facilitators emerged, including improved knowledge, increased accessibility, and addressing discrimination. All stakeholder groups voted for lack of awareness and knowledge as the priority barrier to address. All co-designed interventions consisted of a multimodal PrEP awareness campaign tailored to Black communities, with an emphasis on Black women’s involvement to foster trust and engagement. However, the workshops produced different approaches, with Black women focusing on community-led initiatives, and HCPs advocating for government-backed, broader strategies despite known distrust of institutions.

**Conclusions:**

This study highlights the importance of co-designing interventions with Black women to address multi-level barriers to PrEP access. It underscores the need for community education, healthcare system reforms, and the inclusion of Black women in decision-making processes to reduce PrEP equity gaps. The co-designed interventions provided a tailored, context-specific strategy that could improve PrEP uptake among Black women in England.

**Supplementary Information:**

The online version contains supplementary material available at 10.1186/s12889-025-23023-5.

## Introduction

In England, Black women face substantial disparities when it comes to HIV outcomes and prevention [1], including in accessing HIV pre-exposure prophylaxis (PrEP), an intervention shown to be highly effective at preventing HIV acquisition [2, 3]. Although Black African women accounted for nearly one-third of new HIV diagnoses first made in England among people who acquired HIV through heterosexual contact in 2023, they only represented 3% of all heterosexual PrEP users [4]. In fact, our previous work showed that Black African women are the most underserved population by PrEP as their number of new HIV diagnoses (a proxy for PrEP need) relative to PrEP users was 278 times smaller than that of white men, followed by Black Caribbean women as the second most underserved population [5]. This stark inequality followed the uncapped commissioning of PrEP in 2020 by the National Health Service (NHS), raising critical questions about structural barriers, and the effectiveness of the current approach to exclusively deliver PrEP in specialist sexual health services (SSHS): a systematic review found that over three-quarters of the studies investigating the barriers to PrEP access in the UK focussed on men who have sex with men (MSM) and that 80% of said barriers were at the patient rather than provider or structural level [6].

HIV research and interventions have historically been shaped by activism, with affected communities demanding meaningful participation in scientific knowledge generation and health policy development and implementation [7]. This approach began with the 1983 Denver Principles manifesto, where people living with HIV rejected being viewed as victims [8], and evolved through the Greater Involvement of People living with HIV/AIDS (GIPA) principles [9, 10]. The GIPA Principles recognised that “*the full involvement and participation* [of people living with HIV] *in the design, planning, implementation and evaluation of programmes is crucial to the development of effective responses to the HIV/AIDS epidemic*” [10]. Similarly, early PrEP effectiveness trials in Cambodia and Cameroon were halted by community activism due to concerns of unethical practices, and insufficient consultation with the research populations [11, 12]. In response, UNAIDS developed its 2011 Good Participatory Practice guidelines for biomedical HIV prevention trials, which require funders, sponsors, and implementers to undertake appropriate stakeholder engagement activities [13].

Despite the NHS’s legal duty to involve the public and patients early in services commissioning and decision-making processes [14, 15], such activities were only undertaken in the final stages of PrEP roll-out in England. This limited engagement with key populations likely contributed to the significant and growing equity gap in PrEP access [5]. Furthermore, while patient, public involvement and engagement (PPIE) activities, a form of “*invited activism*” [16, 17], are increasingly mandated in healthcare research and services [13, 15, 18], evidence supporting their impact on healthcare quality improvement is mixed and inconclusive [19–21]. A systematic review found that collaborative elements of PPIE sometimes overshadow the quality of studies and knowledge generation [21]. Little evidence assesses the added value of inclusive research compared to research done without the involvement of patients or the public [22]. Poor quality of evidence and confusion over what constitutes PPIE, especially in sexual health, contributes to this issue, with qualitative research often mistakenly labelled as PPIE, including activities undertaken only after key decisions were made [23, 24].

This study therefore has dual aims: to improve the equitable delivery of PrEP in England by identifying modifiable barriers and facilitators to PrEP access among Black women, and to investigate effective co-design practices for developing interventions to address these challenges. By focusing on Black women – the population most underserved by PrEP relative to need – and using a structured two-phase approach combining focus groups and co-design workshops, we seek to develop tailored, context-specific interventions while simultaneously evaluating the impact of different stakeholder involvement strategies on intervention development.

## Methods

We conducted a qualitative study in two phases: Phase 1 involved focus group (FG) discussions exploring barriers and facilitators to PrEP access experienced by Black women in England; Phase 2 used this data in co-design workshops to develop new interventions to address these factors. In this study, we used a broad definition of PrEP access, drawing on Penchansky and Thomas’s theory of healthcare access that extends beyond simply obtaining a PrEP prescription [25]. Applying the theory to our investigation, true access encompasses the entire journey to effective PrEP use, including overcoming barriers to initiation, adherence and maintenance – all essential components of the PrEP care continuum.

### Phase 1 – Focus group discussions

#### Study design

We chose FGs for Phase 1 of this project because they offered special insight into the various stakeholder dynamics involved in PrEP delivery and access, and allowed participants to build upon each other’s input [26, 27]. Group interviews also enabled consensus-building to identify the most important factors according to Black women and other stakeholders [27, 28], forming the basis of the co-design workshops of Phase 2.

We held three series of FGs, each representing key stakeholder profiles and perspectives important in delivering PrEP in England and understanding why Black women were the most underserved:

- Black women public members represented the potential end users and their individual experiences of SSHS and PrEP access, as our previous work identified them as the population most underserved by PrEP delivery [5];
- Healthcare professionals (HCP) included clinicians, nurses, and Local Authority (LA) commissioners responsible for delivering and/or commissioning SSHS and PrEP in England, representing provider and structural-level perspectives.
- A mixed group of Black women and HCPs represented the ideal PPIE strategy, acknowledging that Black women’s lived experiences are as valuable as professional expertise in understanding the barriers and facilitators to PrEP access, as per the UNAIDS guidelines on good participatory practices [13].

Black women had to be over 18 years old to be eligible for participation and have a negative or unknown HIV status (as PrEP is not for people living with HIV). Black women also had to reside in England, be fluent in English and could be past, current or never users of PrEP. Any HCP was eligible if they were involved in the commissioning and/or delivery of PrEP in England and fluent in English.

There was no prior relationship between the research team and the study participants before study commencement, so recruitment was done in partnership with various community-based organisations for Black women and professional networks related to sexual health and HIV prevention for HCPs. For Black women, that involved sharing recruitment posters and study information with community organisations, who then cascaded these materials through their established networks via email, WhatsApp groups and social media. For HCPs, recruitment was primarily done through professional networks including the British Association for Sexual Health and HIV (BASHH), the British HIV Association (BHIVA) and the English HIV and Sexual Health Commissioners Group (EHSHCG). Additionally, recruitment posters were disseminated across academic networks in national newsletters, social media and specialised research recruitment websites like Call For Participants and People in Research. Due to the slow response rate and limited study timelines, anyone who fulfilled the inclusion and exclusion criteria was invited to participate.

Three FGs were conducted for each stakeholder group (for a total of 9 FGs) and they lasted between 1 h 12 min and 1 h 28 min and participants were compensated £50. Participants were asked during recruitment about their preference to join either a single-stakeholder (Black women-only or HCP-only) or mixed-stakeholder FG, and we accommodated these preferences whenever possible based on their availability while balancing FG sizes. As a result, the number of attendees for each FG varied between four to seven participants for a total of 45 participants: 15 participants in the Black women-only group, 16 in the HCP-only FGs and 14 in the mixed stakeholder FG series (Four black women and 10 HCPs). The lead author (FC, a mixed-race man) moderated all FGs to ensure consistency across the study as this was part of his PhD project. For discussions that involved Black women, one of the Black women peer researchers (WT or FA) co-moderated with him to facilitate race and gender concordance with participants. For discussions that exclusively involved HCPs, he co-moderated with another academic team member experienced in qualitative interviews (KKM or APR, both men). Due to participants’ preferences, all FGs were held online via a College-accredited Zoom account between January 2023 and March 2023. The FGs explored topics such as behavioural approaches to managing HIV acquisition risk amongst Black women in England, their views of PrEP, and factors perceived to hinder and/or facilitate access to PrEP in that population. The semi-structured topic guide used to moderate the FGs (Supplementary Information File 1) was developed specifically for this study by incorporating the findings from a systematic review of the barriers and facilitators to PrEP access in the UK and were reviewed by the peer researchers [6]. Conversations were recorded using the meeting platform record function and transcribed verbatim for analysis.

#### Analysis

The first author (FC), the peer researchers (WT or FA) and KKM analysed the FG transcripts together via thematic framework analysis using both deductive and inductive approaches. The initial codebook was developed based on a systematic review [6] and the FG topic guide. A third of the transcripts were reviewed to incorporate emerging themes, as this subset was sufficient to reach thematic saturation with no new significant themes emerging from additional transcripts. All nine transcripts were then coded with the updated codebook, with only minimal refinements needed as coding progressed, further confirming saturation. A final review of every transcript ensured all relevant quotes were appropriately coded. The thematic framework was derived from the finalised codebook, categorizing codes into themes based on their relationships (Supplementary Information File 2). NVivo 12, a Computer Assisted Qualitative Data Management Software, was used to facilitate the study data handling during the analysis.

The findings were further analysed using the Capability, Opportunity, Motivation and Behaviour (COM-B) model of behaviour change to gain insights into the modifiable factors affecting PrEP access among Black women in England [29] (Table 1). When applying the COM-B framework, we found that a few themes could be categorised into two components of the framework and therefore assigned them accordingly as others have done in the past [30]: for example, cultural attitudes and stigma were co-assigned to both social opportunity and automatic motivation, as these attitudes originated from the societal environment but were subsequently internalised by Black women, affecting their motivation to use PrEP.

**Table 1:** The COM-B model, a framework for understanding behaviour (Adapted from Michie et al.) [29]. The capability and opportunity components of the COM-B model can influence one’s motivation and all three components of the model interact with the behaviour being studied

COM-B components	COM-B sub- components	Sub-components description
**Capability** – *a person’s capabilities to engage in a behaviour*	Physical capabilities	Physical skills, stamina and/or strength
	Psychological capabilities	Psychological skills, stamina and/or strength to engage in the necessary mental processes (including knowledge)
**Opportunity** – *External factors that facilitate behaviour enactment*	Physical opportunity	Opportunities afforded by the environment (e.g. time, financial resources and location)
	Social opportunity	Opportunities afforded by interpersonal influences, societal cues and cultural norms
**Motivation** – *Influenced by the previous two components (i.e. capability and opportunity)*	Reflective motivation	Analytical decision-making based on experience or knowledge and making plans
	Automatic motivation	Desires, impulses and emotional responses

### Phase 2 – Intervention co-design workshops

#### Study design

In Phase 2, we conducted workshops to simulate co-design activities meant to be undertaken in the commissioning stages of NHS interventions in England. These workshops aimed to investigate effective co-design practices for developing interventions to address modifiable barriers and facilitators to PrEP among Black women. One 3-h workshop was held for each stakeholder combination used in Phase 1 (for a total of 3 workshops) where every FG attendee was invited to participate in the workshop of their respective series and compensated £75 for participating.

The workshops were conducted in the last two weeks of March 2023: the first author (FC) and one of the peer researchers (FA or WT) co-facilitated in-person workshops for the streams involving Black women public members due to their collaborative nature, while FC and APR co-facilitated the HCP-only workshop online to accommodate participants’ work schedules. The date and time of the workshops were decided based on the participants’ availability. We adapted the Behaviour Change Wheel (BCW) as a guiding framework to support participants in co-designing their intervention [29, 31]. Notes and end products (i.e. newly designed interventions) were collected for analysis.

First, as per the BCW guiding framework, participants reviewed all modifiable factors previously voted as most important in their respective preceding FGs and selected which one to co-design an intervention for through consensus. Then they were asked to list all stakeholders involved in or with a stake in the delivery of PrEP in England, including those not sufficiently involved. Next, participants were guided on how to co-design their intervention using the adapted BCW handouts (Supplementary Information File 3): They discussed which of the nine intervention *functions* listed in the BCW their intervention could adopt and which *type* of intervention could deliver such function(s) [29, 31]. Finally, the participants designed the content of the intervention based on the agreed intervention function(s) and policy type (i.e. who, what, when, where, how, with whom).

#### Analysis

Due to the lack of well-developed co-design impact assessment tools, we used the APEASE (Affordability, Practicability, Effectiveness/cost-effectiveness, Acceptability, Side-effects/safety, Equity) criteria to assess the quality of each intervention as it is integrated with the BCW [29, 32] (Table 2). Each criterion was assessed on a predefined numeric scale and graded according to available scientific evidence on similar interventions and previous implementation experiences. While we would normally commission additional studies to address any lack of evidence or uncertainties, we instead considered the contextual factors specific to PrEP delivery in England and anecdotal reports due to the project timelines: FC performed the initial assessment and the co-authors reviewed the grading. This grading system enabled the standardisation needed to compare which co-design practice developed the most effective intervention to address PrEP inequity for Black women in England.

**Table 2:** The APEASE criteria for evaluating interventions and their components. This grading system provided the standardisation needed to determine whether PPIE and inclusive co-design practices (i.e. bringing Black women into policy decision-making) developed an intervention better suited to address PrEP access inequities compared to the standard professional-only design. Adapted from West et al. [32]

**APEASE criteria**	**Criteria description**	**Numeral grading system**^a^
Affordability	How far can it be afforded when delivered at the scale intended? Can the necessary budget be found for it?	0 to 10
Practicality	Can it be implemented at scale as designed within the intended context, material and human resources?	0 to 10
Effectiveness & cost-effectiveness	How effective is the intervention in achieving the desired objectives in the target population? How far will it reach the intended target group and how large an effect will it have on those reached?	0 to 10
Acceptability	How far is it acceptable to all key stakeholders?	0 to 10
Side-effects & safety	What unintended adverse or beneficial outcomes might it lead to? How important are they and what is the likelihood that they will occur?	-5 to + 5
Equity	How far does it increase or decrease differences between advantaged and disadvantaged sectors of society?	-5 to + 5

^a^Grading involves an element of judgement but should rely on evidence where available to make such a judgment

### Peer researchers

We involved two Black women public members as peer researchers (FA and WT), whom we trained in qualitative research. They participated in most of the research cycle including co-designing the study, obtaining additional funds, co-moderating activities, co-analysing data and jointly presenting results at conferences. During these processes, any disagreements between the peer researchers and the first author were reconciled through open discussion and a subsequent vote.

## Results

### Focus groups

#### Study participants

The recruitment of Black women participants was biased towards middle-aged women living in London: Among Black women-only FG participants, 60% were between 35 and 49 years old (*n* = 9/15), and 80% lived in London (*n* = 12/15) (Table 3). Only 4 of 14 participants in the mixed stakeholder group were Black women public members with the rest being HCPs.

**Table 3:** Summary demographic characteristics of participants completing the FG discussions

**FG Series**	**Participant role**		**Ethnicity**	**Gender**	**Age group**	**Region**^a^** of residence/work**
**Black women-only FGs**	Black women public members (*n* = 15)		Black African (*n* = 5) Black British (*n* = 2) Black Caribbean (*n* = 7) Black other (*n* = 1)	Female (*n* = 15)	18–24 (*n* = 1) 25–34 (*n* = 5) 35–49 (*n* = 9)	East (*n* = 1) London (*n* = 12) Midlands (*n* = 1) North West (*n* = 1)
**HCP-only FGs**	Consultant in Public Health (*n* = 1) PrEP programme lead (*n* = 1) LA commissioner (*n* = 5) Public Health Specialist (*n* = 1) Senior Health Protection Nurse (*n* = 1) SSHS nurse (*n* = 3) SSHS pharmacist (*n* = 1) SSHS clinician (*n* = 2) Outreach Education (*n* = 1)		Black (*n* = 2) White (*n* = 14)	Female (*n* = 10) Male (*n* = 6)	25–34 (*n* = 3) 35–49 (*n* = 5) 50–64 (*n* = 6) Missing (*n* = 2)	London (*n* = 1) Midlands (*n* = 1) North East and Yorkshire (*n* = 3) North West (*n* = 1) South West (*n* = 6) South East (*n* = 2) Missing (*n* = 2)
**Mixed stakeholder FGs**	Black women public members (*n* = 4)		Black African (*n* = 3) Black British (*n* = 1)	Female (*n* = 4)	18–24 (*n* = 1) 25–34 (*n* = 2) 35–49 (*n* = 1)	London (*n* = 3) North West (*n* = 1)
	HCPs (*n* = 10)	SSHS clinician (*n* = 5) SSHS nurse (*n* = 1) LA commissioner (*n* = 2) Public Health Programme Manager (*n* = 1) Sexual health advisor (*n* = 1)	Arab (*n* = 1) Black (*n* = 1) Mixed (*n* = 1) White (*n* = 7)	Female (*n* = 9) Male (*n* = 1)	25–34 (*n* = 1) 35–49 (*n* = 6) 50–64 (*n* = 3)	East (*n* = 1) London (*n* = 6) Midlands (*n* = 1) North East and Yorkshire (*n* = 1) South East (*n* = 1)

*Abbreviations*: *FG* Focus Group, *HCP* Healthcare Professional, *PrEP* Pre-Exposure Prophylaxis, *LA* Local Authority, *SSHS* Specialist Sexual Health Services, *NHS* National Health Service

^a^NHS regions

HCPs were recruited from across England and included a range of professional and sociodemographic backgrounds: over 80% of HCPs were white (*n* = 21/26), and one-third were 50 years old or older (*n* = 9/26), while all Black women participants were under the age of 50 years old (Table 3).

#### Barriers to PrEP access

**Fig. 1:**
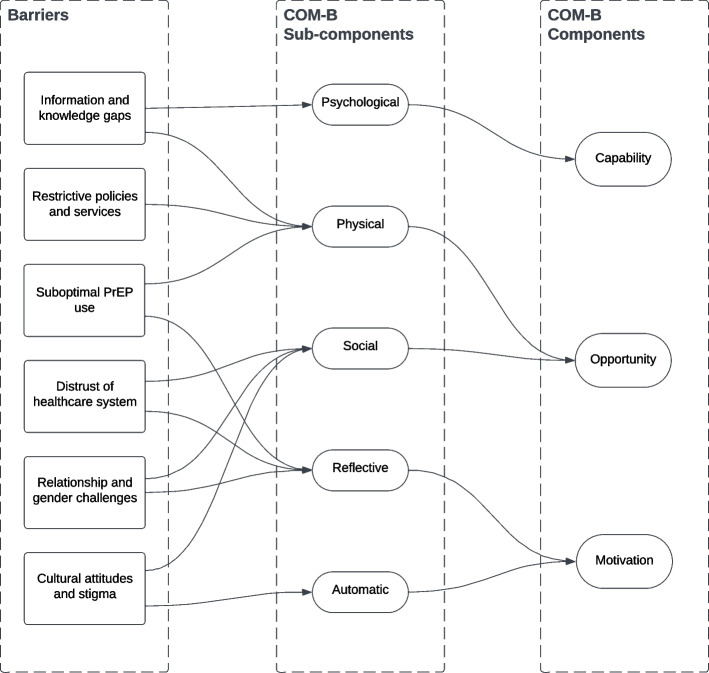
Schematics of the barriers mapped onto the COM-B model of behaviour change. The barriers were mapped to all by one of the sub-components of the COM-B model and importantly, all factors were mapped at least once to the opportunity component (i.e. provider and system-level)

Participants across stakeholder groups highlighted six barriers to PrEP access faced by Black women in England (Fig. 1): Information and knowledge gaps, restrictive policies and services, cultural attitudes and stigma, distrust of the healthcare system, relationship and gender challenges, and suboptimal PrEP use. These barriers were mapped to five out of six of the COM-B sub-components (Fig. 1): psychological capability, physical opportunity, social opportunity, reflective motivation, and automatic motivation.

1. Information and knowledge gaps

Participants across stakeholder groups identified the lack of awareness and knowledge about HIV and PrEP among Black women as the most important barrier (Supplementary Information File 4). This barrier was linked to a combination of individual-level lack of HIV and PrEP knowledge amongst Black women and insufficient information dissemination to Black communities from providers and the NHS. Many participants across stakeholder groupings highlighted this knowledge gap e.g. Misconceptions such as PrEP being exclusively for MSM were prevalent. Consequently, many Black women did not perceive themselves as at risk of HIV acquisition and thus did not consider PrEP a viable prevention option. This knowledge gap was further exacerbated by limited public health campaigns and education on PrEP specifically aimed at Black women. As such, this barrier was co-assigned to the psychological capability and physical opportunity sub-components of the COM-B model (Fig. 1):

“I think there’s like, there’s nowhere to find out about PrEP as a Black woman, because it doesn’t feel like it’s targeted to me. […] And you need to know about it. Right. So I’m not hearing that.” – Participant in Black women-only FG3.

2. Restrictive policies and services

The restrictive policies and services theme represented the strongest provider and system-level barrier, hence its sole assignment to the physical opportunity sub-component of the COM-B model (Fig. 1). All FG discussions agreed that the sole availability of PrEP in SSHS and a lack of an integrated healthcare system represented a major structural barrier to PrEP access for Black women due to the stigma they attached to using SSHS and their preference to attend non-SSHS for their Sexual and Reproductive Health needs:

"One approach or one size fits all isn't ever the right model, and doesn't address inequalities or increase equity. So I think the lesson I've taken from that is the need to tailor services for different people, whether that's different communities, or actually different individuals, rather than expecting everybody to access one model of service provision." – Participant in HCP-only FG2.

Moreover, the lack of prioritisation of sexual health from policymakers resulted in underfunding of SSHS in some regions and limited access, creating disparities based on geographic location. Additionally, HCP-only and mixed-stakeholder FG participants agreed that the current PrEP eligibility criteria overly focused on MSM and MSM-associated behaviours and overlooked women's HIV risk. Black women in mixed-stakeholder FGs noted the inconsistencies of the eligibility criteria as they agreed that they did not have to practice condomless anal sex to be at risk of HIV acquisition: one woman reflected how “*anyone that's engaging in sex is at risk”* of HIV acquisition, no matter how many or type of sexual partners they might have.

Finally, there was a recurrent view in FGs involving HCPs that the process of getting a PrEP prescription was overly complex with “*too many hoops*” to jump through (e.g. the requirement for previous at-risk behaviours). Participants went further by admitting that the gatekeeping role that the clinician plays in deciding whether their patient is at risk of HIV acquisition to “*bless them with the gift of PrEP*” is paternalistic and problematic in this day and age.

3. Cultural attitudes and stigma

Cultural^1^ and community-level stigma (including religious beliefs, homophobia, HIV and PrEP stigma, and taboos around sex) was another significant barrier leading to underestimation of HIV risk and lack of PrEP willingness among Black women. FG participants discussed how Black people internalised the stigma that originated from their societal and community environments, hence the co-assignment of this barrier to the social opportunity and automatic motivation sub-components of the COM-B model (Fig. 1). Faith was an important sub-theme as Black women participants often contextualised stigmatising views towards sex, HIV and PrEP within their community because of traditional religious beliefs:

“You have to have a really open-minded reverend/pastor, church sister, brother, because I cannot see them wanting to lead that. Because I think their doctrine is like, you know, we're still with a lot of them who are very traditional: So abstinence, no sex before marriage. When really, you know, it's a modern world. And we're moving away from that. I mean, obviously, I respect everyone's religion, but these are the realities, we need to accept the realities because our church brother and our church sister are having unprotected sex” – Participant in Black women-only FG2.

That prevented open discussions about sexual health as it was widely acknowledged that PrEP would be associated with promiscuity, causing resistance among Black women, in fear of judgment. This barrier was further compounded by the emphasis on privacy in Black communities, especially for health-related matters, as Black women participants showed concerns about being seen accessing SSHS or being cared for by HCPs of the same background.

Finally, while only discussed a few times across streams, the FGs also revealed entrenched homophobia within Black communities and wider society. This resulted in beliefs that HIV was predominantly a concern for gay men, fostering ignorance among Black heterosexual men and women about their own risk.

4. Distrust of the healthcare system

Distrust of the healthcare system and institutions was a recurring barrier to PrEP access among Black women and one of the most important factors, according to the FG participants (Supplementary Information File 4). This distrust and scepticism of the NHS and HCPs stemmed from previous negative experiences and known healthcare outcome inequalities caused by institutional racism and intersectional prejudice in the healthcare system. This barrier was co-assigned to the social opportunity and reflective motivation sub-components of the COM-B model as providers, and the wider system perpetuated institutional racism and intersectional prejudice, which was internalised by Black women, resulting in medical de-prioritisation (Fig. 1). Participants across all FG series agreed that the NHS is not designed to address Black women's needs, as their “*bodies and experiences are not seen as worthy or as valuable*”, as evidenced by past and current historical injustices. Black women participants reported experiencing a “*double whammy”* of sexism and racism and explained how they were subjected to certain expectations and negative stereotypes when accessing healthcare: they described several instances when they were disrespected, sexualised or had their healthcare needs dismissed by HCPs.

Meanwhile, despite acknowledging this barrier, HCPs often lacked self-reflection about how their own practice within the healthcare system might contribute towards institutional racism. The HCP-only discussions downplayed the role of institutional racism within the NHS as only a few HCPs named racism as such despite the consensus that Black women experience inequity and discrimination. In fact, one HCP even questioned whether ethnicity was the factor at play:

“Is there another underpinning element to it that is more accurate at assessing why these people struggle to access services, rather than just say the colour of the skin?” – Participant in HCP-only FG1.

5. Relationship and gender challenges

All streams discussed the relationship and gender challenges hindering HIV prevention and PrEP access for Black women as rooted in power imbalances within relationships and gender roles: participants across stakeholder groupings reported how some Black women they knew wrongly assumed they were in monogamous relationships despite known infidelity and concerns about STIs. This represented a significant cognitive dissonance as participants discussed how common this was within Black communities. Black women attendees emphasised the role that their communities’ cultural beliefs played in fostering ignorant attitudes toward how Black Caribbean and African men approached sexual health and relationships:

“You might be the best you can be as who you are, your partner’s who your partner is. And still coming from an ignorant society where the men, Caribbean and African men don't really believe in monogamy.” – Participant in Black women-only FG1.

Indeed, HCPs noted their struggle to address and discuss those situations with their Black women patients in a sensitive and culturally appropriate manner. Additionally, participants across groups highlighted how some Black women were unable to negotiate safe sex practices within their relationships and how they might fear using PrEP at home due to controlling partners. This barrier was co-assigned to the social opportunity and reflective motivation sub-components of the COM-B model due to the social gender norms that Black women had to manage within their relationships (Fig. 1).

6. Suboptimal PrEP use

The final barrier identified in the FG discussions was suboptimal PrEP use due to challenges in initiating and adhering to PrEP and reservations about the prophylaxis itself. As this barrier resulted from the lack of PrEP knowledge and environmental factors that hinder Black women public members from using it effectively, it was co-assigned to the physical opportunity and reflective motivation components of the COM-B model (Fig. 1). However, important differences arose from the different stakeholder groupings, which spoke to the participants’ respective backgrounds. HCP participants focused on structural issues like limited access to SSHS and the exclusion of incarcerated women due to rigid provision systems. They agreed that the exclusive commissioning of PrEP in SSHS leaves women from some communities behind as “*people have to travel a long distance to get to a sexual health service*” and might not “*have got the time or the money to be able to travel*”. Meanwhile, Black women participants highlighted personal reservations rooted in negative experiences with hormonal contraceptives, concerns about side effects, pill fatigue, busy schedules, and the lack of alternative PrEP modalities like injectables.

#### Facilitators to PrEP access

**Fig. 2:**
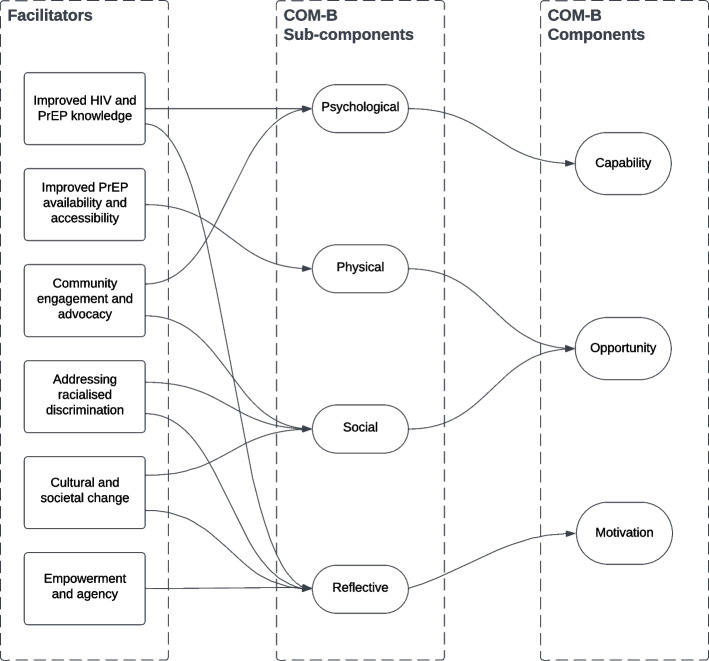
Facilitators mapped onto the COM-B model of behaviour change. Facilitators were mapped to four of the six sub-components of the COM-B model

Participants across stakeholder groups discussed six facilitators to PrEP access to Black women in England (Fig. 2): Improved HIV and PrEP knowledge, improved PrEP availability and accessibility, community engagement and advocacy, addressing racialised discrimination, cultural and societal change, and empowerment and agency. These facilitators were mapped to four out of the six COM-B model sub-components (Fig. 2): psychological capability, physical opportunity, social opportunity, and reflective motivation.

Many of those facilitators could operate as a direct counterbalance to the barriers explored above: Improving HIV and PrEP knowledge through targeted, culturally sensitive educational campaigns was voted as the most important facilitator to PrEP access among Black women (Supplementary Information File 5). Improving PrEP availability by expanding PrEP commissioning beyond SSHS to more accessible settings like general practices and pharmacies was a facilitator that could counterbalance the barrier of restrictive policies and services. This would address logistical barriers and stigma associated with attending SSHS and was therefore voted as the second most important by participants. Similarly, the barrier of distrust of the healthcare system can be tackled by addressing racialised discrimination via culturally competent HCPs, increased diversity of HCP representation, and co-design of services with Black communities. In turn, this would rebuild Black women’s trust in HCPs and the wider healthcare system.

The other facilitators were not identified to directly counterbalance the other barriers but spoke to the role that Black women can have in addressing PrEP inequity in England. Participants emphasised the importance of community engagement, as trusted community figures could play a vital role in disseminating information on HIV and advocating PrEP use. This facilitator was co-assigned to the psychological capability and social opportunity sub-components of the COM-B model as it leveraged trusted community peers to promote trust, knowledge and PrEP uptake (Fig. 2):

“I believe it has to be explained in the community by people they can relate to, not like, I’m not being racist, but these white people, they just want to test us or decided that they just want to try something, or they want to use us. Sometimes this is the mentality that we have.” – Black woman participant in mixed-stakeholder FG3.

This aligns with the facilitator of promoting empowerment and agency to support Black women in asserting their autonomy over their bodies and sexual health and was therefore mapped to the reflective motivation sub-component of the COM-B model (Fig. 2). All stakeholder discussions agreed that PrEP should be presented to Black women as an empowering tool to control and owe their bodies, sexual health and HIV status and promote their overall health and well-being. Black women participants emphasised that this was particularly relevant for situations when they could not negotiate safe sex practices such as condom use. Furthermore, mixed stakeholder participants agreed that PrEP should be promoted to Black women with a sex-positive mindset as it would allow them to have a more “*enjoyable sex life, and a safer and more comfortable empowered sex life*”.

Lastly, participants across stakeholder groupings supported long-term societal shifts in cultural attitudes towards sexual health and HIV prevention as it could create a more supportive environment for PrEP access. As this would entail the normalisation of sexual health discussions, destigmatising HIV and reframing PrEP perceptions at the individual and societal levels, this facilitator was mapped to the reflective motivation and social opportunity sub-components of the COM-B model (Fig. 2). While all participants agreed that such conversations should be integral to every point of the life course, Black women participants pointed out that the younger generations could play a pivotal role in facilitating wider social change as they are already undergoing a shift in their approach to sex and relationships:

“I think a lot about like how my peers and I talk about just the way in which relationships and sexual relationships are changing, and are quite different for people who are younger, who are experimenting with things like multiple partner relationships.” – Black woman participant in mixed-stakeholder FG2.

### Co-design workshops

Four out of the 14 mixed-stakeholder FG participants joined the co-design workshop, which included two Black women, one SSHS clinician and one LA commissioner. Seven out of the 15 Black women-only FG participants (all of whom lived in London), and six of the 17 HCP-only FG participants (two LA commissioners, one SSHS nurse, one PrEP programme lead, one Public Health lead and one SSHS pharmacist) attended their respective co-design workshop.

#### Selection of modifiable factors

All workshops agreed that tackling PrEP inequity for Black women in England should follow a multi-intervention approach to address multiple barriers. However, due to the limited time available for these co-design activities, participants were directed to pick a single factor and intervention throughout the workshop. All three workshop streams agreed the lack of awareness and understanding of HIV and PrEP among Black women was the most important barrier that needed to be addressed, as per the FG consensus exercise (Supplementary Information file 4). They recognised that improving HIV and PrEP knowledge would be the most impactful, as it could address multiple barriers simultaneously. This included providing Black women with the resources to appropriately assess their risk of HIV acquisition, reducing stigma and promoting self-agency. All workshops discussed the need for this facilitator to be leveraged specifically for Black women against the backdrop of institutional racism within the healthcare system as Black spaces and women were considered left out by PrEP promotional materials.

#### Stakeholder mapping

Participants identified a broad range of stakeholders involved in PrEP delivery, including HCPs, pharmaceutical companies, and institutions like the NHS and the education system. Notably, the workshops involving HCPs (mixed and HCP-only) listed more HCPs and policymakers than the Black women-only workshop. The latter could not identify some of the key stakeholders involved in commissioning PrEP and writing PrEP guidelines (Supplementary Information File 6).

All workshops with Black women participants highlighted the importance of leveraging online sexual health providers and educational institutions to introduce and promote HIV and PrEP knowledge, which the HCP-only workshop did not discuss: while online services could provide PrEP information material and link users to PrEP services, Relationships and Sex Education classes in schools could be important settings to introduce HIV and PrEP knowledge. Additionally, workshops involving Black women acknowledged that community organisations of all backgrounds were under-utilised stakeholders to promote HIV knowledge and PrEP access; while the HCP-only workshop mostly focussed on racially minoritised community and HIV organisations.

#### Intervention design

All workshops agreed that the intervention should aim to *educate* Black women on HIV and the preventions available against it and *convince* them that PrEP is a tool for self-agency. This could be done in part by having Black women PrEP users *lead by example* by talking about their experience of PrEP and inducing positive feelings towards the prevention. Additionally, the mixed-stakeholder workshop participants thought that the intervention could *incentivise and motivate* Black women to use PrEP by creating the expectation of more pleasurable and satisfactory sexual relations. As such, the participants across workshops settled on an intervention focused on *public health communication/marketing campaigns* to increase HIV and PrEP knowledge.

On that basis, all workshops co-designed interventions with similar core principles: The proposed campaigns were multimodal, incorporating national media and community-level outreach that leveraged trusted Black women as the faces of the campaigns to build credibility and trust within Black communities. These women would lead in disseminating information through television, radio, social media, and in-person community events, such as roadshows in places frequented by Black women (e.g. hair salons, community centres, schools, and festivals). Participants agreed that this combination of national mass media and local engagement was essential to reach the diverse demographic of Black women across the country. Another core principle was the need to assess and evaluate the effectiveness of the intervention by measuring its impact on HIV and PrEP knowledge among Black women via quantitative metrics such as changes in attitudes.

Participants of the HCP-only workshop insisted on co-designing a second intervention aimed at improving HIV and PrEP knowledge among HCPs. This intervention focused on updating the current PrEP clinical guidelines to move away from MSM-centric approaches to PrEP prescribing and provide support to HCPs on how to discuss PrEP with Black women in a culturally sensitive manner. This guideline-based intervention aimed to ensure that Black women are consistently assessed for eligibility and introduced to PrEP during healthcare appointments.

#### Differences between workshops

While all workshops agreed on the core approach of raising awareness through public health campaigns, there were notable differences in the details of the interventions that reflected the stakeholders’ backgrounds. The Black women-only workshop emphasised the need for a grassroot initiative for community empowerment and proposed creating a Black women-led organisation to manage and lead the campaign. The Black women-only workshop intervention design had an additional component that involved community training events for women running businesses and spaces dedicated to Black women for them to learn how to share HIV and PrEP knowledge with their clients (i.e. peer community champions). Notably, they did not discuss the involvement of HCPs and other policymakers, nor did they talk about funding and piloting the campaign. In contrast, the HCP-only workshop intervention emphasised the need for broader systemic support, including government funding and integration of the campaign into the wider national HIV elimination strategy: HCPs discussed the Hepatitis C elimination strategy as the perfect example of coordinated investments and regular evaluations and revisions through specific key performance indicators. However, in their “*coalition of the willing*”, they only listed Black women in the public-facing role.

Meanwhile, the mixed workshop took a more collaborative approach that brought together the expertise of Black women public members, policymakers and established HIV organisations. This proposed partnership aimed to enhance the campaign’s credibility with Black audiences.

#### APEASE evaluation

The public health campaign interventions were considered *effective* based on previous public health mass media HIV and STI campaigns that showed better HIV knowledge and sustained effects on STI prevention [33, 34] (Table 4). However, previous studies were not done for the population of interest (i.e. Black women), hence the slightly above-average grading of the public health campaign intervention on the effectiveness criteria.

**Table 4:** Evaluation of the interventions and their components using the APEASE criteria. The APEASE criteria are meant to enable the comparison between interventions and determine which one was most effective in increasing HIV and PrEP awareness and knowledge among Black women in England

APEASE criteria (Numeral grading system)	PrEP promotional campaign intervention	Guideline intervention (HCP-only workshop)
**Affordability** (0 to 10)	3	10
**Practicality** (0 to 10)	6	10
**Effectiveness** (0 to 10)	6	7
**Acceptability** (0 to 10)	10	7
**Side-effects & safety** (-5 to + 5)	+ 5	+ 5
**Equity** (-5 to + 5)	+ 5	+ 5
**Total**	35	44

Those interventions were also considered highly *acceptable* as the workshop participants represented a wide range of stakeholders involved in PrEP and they all came to design similar promotional campaigns, hence the full marks for that criterion. However, the public health campaign interventions scored low on *affordability* due to the high cost of implementing national mass media campaigns [35, 36]: While there were no estimates publicly available on how much an HIV public health educational campaign would cost, the budget of an English national mass media smoking cessation campaign financed by the Central government was in the millions of pounds over a decade ago [35].

While the in-person and social media aspect of the campaigns would allow to specifically target Black women to increase their knowledge of HIV and PrEP, the national-level mass media side of the intervention (i.e. television, radio and press) only allows for limited targeting, hence the above average score on *practicality*. Additionally, such campaigns should not have *side-effects*, as they would promote HIV and PrEP knowledge in anyone who comes across it and should address the issues of PrEP use *inequity* in England.

In contrast, the additional guideline intervention designed by the HCP-only workshop was both highly *affordable* and *practical*, given that it could be undertaken by BHIVA and its HCP members and integrated into existing SSHS at minimal cost (Table 4). The intervention was also assessed as *effective* at retraining HCPs to assess all their SSHS patients for PrEP eligibility. However, it did not reach full marks due to years of MSM-centric PrEP prescribing and changing this preconceived notion among HCPs might take time, which is also reflected in the *acceptability* criteria. Like the PrEP campaign design, the updated guidelines should not cause *safety* or *inequity* concerns and should in fact address them.

## Discussion

This study used multi-stakeholder FGs and co-design workshops to explore the modifiable barriers and facilitators to PrEP access experienced by Black women in England and develop interventions to address one of those modifiable factors. It also explored the impact of involving potential service users in the decision-making process of intervention design compared to traditional, professional-only decision-making.

The COM-B theory of behaviour change revealed the complex interplay between the individual capabilities (e.g. PrEP knowledge), provider and systemic opportunities (e.g. PrEP accessibility and distrust of the healthcare system) and their impact on Black women’s motivation to use PrEP. The lack of HIV and PrEP knowledge amongst Black women in England was identified as the most important barrier across stakeholders, which reflects previous findings in the UK [6, 37], the WHO European region [38], the USA [39] and France [40]. Consequently, all workshops co-designed interventions that targeted this barrier via a multimodal public health campaign at the national and community levels. While all workshops acknowledged that addressing major structural barriers like restrictive commissioning and NHS institutional racism would likely have greater impact, they pragmatically chose to focus on improving HIV and PrEP knowledge as a more immediately achievable goal within existing political constraints.

However, the co-designed intervention reflected the background and expertise of the respective stakeholders who produced them. While the Black women-only workshop emphasised community-led efforts to trust-building, it notably excluded HCPs and political involvement – reflecting on Black women’s deep-rooted institutional distrust while paradoxically demonstrating their lack of institutional knowledge regarding sustainable financing, piloting, and implementation requirements. Meanwhile, the HCP-only workshop focused on government involvement and institutional strategies (including a second intervention addressing restrictive guidelines) but showed limited community insights by relying on institutions seldom trusted by Black communities. In turn, this design inadvertently replicated tokenistic approaches by including Black women only in public-facing roles. Finally, the mixed workshop emphasized the need for collaboration between communities and trusted institutions to foster trust and engagement with the target population. Unfortunately, the limited functionality of the APEASE criteria could not conclusively ascertain which co-design strategy developed the most effective intervention without piloting the respective intervention.

Our study had several limitations including the recruitment of only HIV-negative English-speaking Black women: This could potentially have excluded women who recently immigrated to England, a sub-population known to face idiosyncratic barriers to the healthcare system [41–43], and women living with HIV who have a unique perspective on missed PrEP opportunities. Furthermore, most Black women participants resided in London: the UK’s biggest urban centre and part of the Fast Track Cities initiative where SSHS are better integrated and funded than the rest of the country [44]. However, Black Londoners accounted for half of the Black population in England and the biggest share of new HIV diagnoses made among Black people in England [45], making their perspectives highly relevant [46].

Additionally, future research will need to include Black men to holistically address PrEP access inequities as they are also underserved by PrEP in England [5]. This will be key to avoiding placing inequity ownership solely on Black women as is often the case in HIV prevention [47], especially as individuals tend to form relationships with others of the same ethnicity [48].

The main limitation of this study is we cannot provide firm evidence on whether one co-design strategy can develop an intervention that is more effective than others as we were unable to pilot them. This adds to the mixed picture of evidence on the impact that PPIE has on outputs aimed at healthcare quality improvement [20–22, 49–52], including in the field of sexual health [19, 53].

However, this perpetuates a false narrative as PPIE holds value beyond improving the quality of interventions: the process itself builds essential and sustainable trust with communities traditionally sceptical of healthcare and research institutions and allows for advances in HIV prevention efforts. The UNAIDS Good Participatory Practice guidelines for biomedical HIV prevention trials state that participatory research rests on promoting positive, collaborative and mutually beneficial relationships [13]. The underlying principles to achieve this trust rely on respect for human rights; mutual understanding achieved through cultural competency; scientific and ethical integrity; transparency through open and honest communication; and community stakeholder autonomy. This reflects the UK National Institute of Health and Care Research’s rationale for public involvement in research [18].

This study exemplifies what trust-building with the research population can achieve: despite Black women participants’ initial discussions of their distrust of the healthcare system and researchers, our sustained rapport built on trust and respect remains as some of the Black women involved have invited us into their community to discuss HIV prevention.

## Conclusions

This study highlights the importance of co-designing interventions with Black women to address the barriers they experience in accessing PrEP. It demonstrates the value of engaging stakeholders to develop tailored solutions, emphasising community education, healthcare system reforms, and inclusivity in decision-making. The co-designed interventions offer promising strategies to reduce PrEP inequities among Black women in England. While further piloting is needed to validate the effectiveness of these approaches, the collaborative process itself was instrumental in fostering trust and shaping interventions that resonate with the target communities. Beyond PrEP access, the co-design methods employed here provide a transferable approach for addressing other healthcare inequities experienced by Black women (such as breast cancer screening, maternal healthcare and mental health services).

## Supplementary Information

Supplementary Material 1: Focus group topic guide. This file is the focus group topic guide used to moderate the group discussions: it was developed based on the findings of a systematic review that investigated the barriers and facilitators to PrEP access in the UK.

Supplementary Material 2: Thematic framework derived from the study codebook summary. Comprehensive thematic framework detailing the themes, sub-themes and their respective descriptions found during the focus group analysis and used to identify the barriers and facilitators to PrEP access in England.

Supplementary Material 3: Workshop co-design hand-outs provided to co-design workshop participants. This file is the detailed workshop materials provided to the study participants to guide them step by step on how to design new interventions to tackle barriers to PrEP access.

Supplementary Material 4: Barriers voted as most important by the FG participants via a consensus-building exercise. Lists of the barriers to PrEP access that were voted as most important by the participants in each focus group stream (mixed stakeholders, HCP-only, and Black women-only), including vote counts and number of participants.

Supplementary Material 5: Facilitators voted as most important by the FG participants via a consensus-building exercise. Lists of the facilitators to PrEP access that were voted as most important by the participants in each focus group stream (mixed stakeholders, HCP-only, and Black women-only), including vote counts and number of participants.

Supplementary Material 6: Stakeholders listed as having a stake in the delivery of PrEP in England. Table categorising the stakeholders identified by each co-design stream as having a stake in PrEP delivery in England. Stakeholders are grouped into five stakeholder categories: HCPs, industry, policy-makers, patients & civil society, and researchers. Italised entries indicate stakeholders identified as currently under-used and/or needing greater involvement in PrEP delivery.

## Data Availability

The datasets generated and/or analysed during the current study are not publicly available due to privacy and ethical restrictions but are available from the corresponding author on reasonable request.

## Abbreviations

AIDS: Acquired Immunodeficiency Syndrome
APEASE: Affordability, Practicability, Effectiveness/cost-effectiveness, Acceptability, Side-effects/safety, Equity
BASHH: British Association for Sexual Health and HIV
BCW: Behaviour Change Wheel
BHIVA: British HIV Association
COM-B: Capability, Opportunity, Motivation and Behaviour
EHSHCG: English HIV and Sexual Health Commissioners Group
FG: Focus Group
GIPA: Greater Involvement of People living with HIV/AIDS
HCP: Healthcare Professional
HIV: Human Immunodeficiency Virus
LA: Local Authority
MSM: Men who have Sex with Men
NHS: National Health Service
PrEP: Pre-Exposure Prophylaxis
PPIE: Patient, Public Involvement and Engagement
SSHS: Specialist Sexual Health Services
STI: Sexually Transmitted Infection
UK: United Kingdom
UNAIDS: The Joint United Nations Program on HIV/AIDS
WHO: World Health Organization

## Footnotes

1 Term used by the Black women participants.
