# Supplementary material for: Co-designing interventions with multiple stakeholders to address barriers and promote equitable access to HIV Pre-Exposure Prophylaxis (PrEP) in Black women in England
Source: BMC Public Health. 2025 May 17;25:1831. doi: 10.1186/s12889-025-23023-5 (PMC12085007; doi:10.1186/s12889-025-23023-5)
Supplement: Supplementary file 1 — Supplementary Material 1: Focus group topic guide. This file is the focus group topic guide used to moderate the group discussions: it was developed based on the findings of a systematic review that investigated the barriers and facilitators to PrEP access in the UK. [file 12889_2025_23023_MOESM1_ESM.pdf]

Facilitators and Barriers to PrEP access in England:

Focus Group Topic Guide

Developed by Flavien Coukan

Warm up questions (5 minutes):

1. What is everyone's thoughts on the current state of sexual health services and HIV prevention in England?
   - *Prompt*: how does that affect the nation's health?

Core questions (20 minutes):

Additional information on PrEP for our public members: *Pre-Exposure Prophylaxis (aka PrEP) is when medicines designed to prevent HIV from replicating are used by HIV-negative people to prevent contracting HIV. Much like taking the contraceptive pill to protect yourself from pregnancy, it involves taking a daily pill to protect yourself from HIV, although researchers are looking into other forms (long-acting injectable, topical gels and microbicides). PrEP works best if people take the medication regularly: Clinical trials and demonstration projects have shown that it is highly effective in preventing HIV acquisition (around 98%) when taken as prescribed. There are some side effects, but this does not affect everyone, and the medicines are generally well tolerated. It is currently available in England via sexual health services only.*

2. When do you think it is appropriate for someone to access PrEP?
   - *Prompt*: What situation?
   - *Optional prompt*: How does that match to the current eligibility guidelines?

Remind everyone of PrEP eligibility if need be:

- *HIV-negative Men who have Sex with Men and transgender women who report recent condomless anal sex and on-going condomless anal sex.*
- *HIV-negative individuals having condomless sex with partners who are living with HIV, unless the partner is virally suppressed (cannot pass it on).*
- *Case by case basis.*

Additional information about how we know Black women are the most underserved: *We performed a quantitative analysis to identify the populations most underserved by the PrEP Impact Trial, a precursor to the national PrEP programme in England, as data from the national programme is not yet available. The analysis was a cross-sectional descriptive analysis of a combination of PrEP prescription data from the PrEP Impact trial and new HIV diagnoses data to describe the distribution of the "PrEP-to-need" ratio (PnR) during the first 29 months recruitment period of the PrEP Impact trial (from October 2017 until February 2020, period prior to the COVID-19 pandemic). The analysis highlighted that Black women have some of the lowest PnR, highlighting how the current delivery model of PrEP significantly underserves their need.*

3. What are your perceptions on how Black women are likely to benefit from PrEP?
    - *Prompt*: Why?

4. Do you think Black women would be open to taking PrEP?
    - *Prompt*: can you explain why?

Facilitators (25 minutes):

5. From whichever capacity you attend this discussion, can you send us two factors that you think could make it *easier* for Black women to access and use HIV PrEP, using the private chat function?

Give everyone a few minutes to send their list and read all factors out loud to the group. Then put the list on screen (or in the chat function).

Prepare the Mentimeter facilitator poll based on the different facilitators submitted. Let the participants know they're going to be prompted to vote.

6. Individually, could you select the 3 most important facilitators for Black women to access and use HIV PrEP?

Present the top 3 facilitators from the polling results.

- *Probe*: Why did people vote for those as these facilitators? What do others think?
- *Check* for agreement and disagreement

7. How can these facilitators happen?
    - *Probe*: How can we take advantage of these facilitators to improve PrEP access and use among Black women?

Barriers (25 minutes):

8. From whichever capacity you attend this discussion, can you send us two factors that you think could make it *harder* for Black women to access and use HIV PrEP, using the private chat function?

Give everyone a few minutes to send their list and read all factors out loud to the group. Then put the list on screen (or in the chat function).

Prepare the Mentimeter barrier poll based on the different barriers submitted. Let the participants know they're going to be prompted to vote.

9. Individually, could you select the 3 most important barriers for Black women to access and use HIV PrEP?

Present the top 3 barriers from the polling results.

- *Probe*: Why did people vote for those as the main barriers? What do others think?
- *Check* for agreement and disagreement

10. How can these barriers be overcome?

- *Probe*: How can we overcome these barriers to improve PrEP access and use among Black women?

Closing down

11. Is there anything you'd like to add?

12. Do you have any questions regarding today or the research?
