# Supplementary material for: Co-designing interventions with multiple stakeholders to address barriers and promote equitable access to HIV Pre-Exposure Prophylaxis (PrEP) in Black women in England
Source: BMC Public Health. 2025 May 17;25:1831. doi: 10.1186/s12889-025-23023-5 (PMC12085007; doi:10.1186/s12889-025-23023-5)
Supplement: Supplementary file 2 — Supplementary Material 2: Thematic framework derived from the study codebook summary. Comprehensive thematic framework detailing the themes, sub-themes and their respective descriptions found during the focus group analysis and used to identify the barriers and facilitators to PrEP access in England. [file 12889_2025_23023_MOESM2_ESM.pdf]

Theme	Theme description	Sub-theme	Sub-theme description
Cultural attitudes and stigma	Cultural and religious stigma surrounding relationships, sex, HIV and PrEP that hinders open conversations and access.	Cultural and religious beliefs	Ingrained cultural norms, values, and religious beliefs within Black communities can contribute to stigma, taboos, secrecy, and reluctance around openly discussing or seeking information about sexual health, including HIV/AIDS and PrEP.
		Homophobia	Homophobia in Black communities fosters beliefs that HIV only affects gay men, and drives Black men to hide their sexual identity: They will publicly be with women to fulfil others' expectations but might still have sex with other men in secret, putting their women partners at risk of HIV acquisition.
		Stigma of HIV	The stigma, shame and trauma associated with HIV within Black communities stem from a lack of understanding of HIV and homophobia. This persistent stigma creates a barrier to accessing SSHS and having open conversations about HIV prevention.
		Stigma of PrEP	Black women fear that they might be ostracised or judged if they talk about PrEP opening or are seen taking PrEP due to assumptions that PrEP is for someone who is promiscuous or high-risk, or that ARV use is only for people living with HIV. This stems from the socially conservative nature of some Black communities and from pre-existing HIV stigma.
		Taboo around sex	This taboo reflects a reluctance and discomfort to discuss SRH (including contraceptives and STIs), often fuelled by cultural norms, religious beliefs, stigmatising perceptions, and the association of sexual discussions with promiscuity or moral decline.
Information and knowledge gaps	Ignorance about HIV and its prevention methods,	Lack of HIV awareness and knowledge	The ignorance and misinformation about HIV and its preventions in Black communities stems from generational

	particularly PrEP, within Black communities.		gaps, cultural stigmas, and gender disparities, and leads to misconceptions, unfounded fears, and missed opportunities for preventative measures.
		Lack of PrEP awareness and knowledge	Insufficient understanding of PrEP in Black communities, and especially Black women, who do not know what PrEP is, how effective it is, its eligibility criteria or its overall benefits, which contributes to lower use of SSHS and PrEP uptake.
		Insufficient information dissemination	There is a lack of targeted education and promotion of PrEP and sexual health resources dedicated to Black communities, particularly Black women, which contributes to lower use of SSHS and PrEP uptake.
		Lack of self-perception of HIV risk	Many Black women do not consider themselves at risk of HIV acquisition due to assumptions about their relationships or partners and a lack of HIV knowledge.
Distrust of healthcare system	Reluctance and scepticism toward the NHS and HCP due to past negative experiences and institutional racism.	Medical de-prioritisation, scepticism & distrust	Black women harbor reluctance, distrust and scepticism toward the healthcare system and HCPs due to cultural stigma, negative personal experiences, and legacy of medical racism, which results in lower engagement with the healthcare system and health information.
		Previous bad experiences within healthcare system	Previous negative interactions, disregard, or discrimination from HCPs or healthcare services shaped reluctance and mistrust among some Black women toward accessing and engaging with health interventions like PrEP.
		Institutional racism in healthcare	Systemic and ingrained racism in healthcare policy, services, research, and interactions negatively impacts Black communities: this manifested through a lack of cultural competence, disregards of Black women’s concerns, and a failure to equitably prioritise and serve racialised minorities.

		Intersectional prejudice in healthcare	Black women face the compounded discrimination in healthcare based on both their race and gender, including disregards of their health concerns, hyper-sexualisation, and assumptions that they have a low risk of HIV acquisition.
Restrictive policies and services	Narrow PrEP eligibility criteria and exclusive access in SSHS overlook women's HIV risk	Lack of integrated healthcare system	The current compartmentalised and siloed NHS services in England impedes Black women's access to PrEP i.e. Lack of coordination across the healthcare ecosystem (primary, secondary and community care).
		Inadequate PrEP eligibility and guidelines	The current criteria and guidelines for who is eligible for PrEP are too narrow and restrictive, as they focus heavily on MSM, which is not suited for women and reflect a paternalistic approach to healthcare with HCPs in control and gatekeeping healthcare.
		PrEP commissioning constraints	Exclusive commissioning of PrEP in SSHS restricts PrEP access Black women as (1) they usually access their SRH needs in community settings, (2) SSHS are stigmatised by the Black community (due to taboo around sex), and (3) inadequate funding of SSHS adds additional strains of services that are already struggling to meet demand.
		Lack of prioritisation of sexual health	Sexual health is not sufficiently prioritised in healthcare policy, funding, and services in England as evident by the delayed national sexual health strategy, limited budgets, and failure to routinely integrate sexual health across services.
Suboptimal PrEP use	The effective use and uptake of PrEP can be challenging due to reservations about the prophylaxis	Suboptimal PrEP uptake and adherence	Some Black women considered PrEP uptake and adherence challenging due to busy schedules, pill fatigue, lack of perceived HIV risk, and side effect concerns.
		PrEP use reservations	These included worries about side effects, and taking a daily pill, the lack of perceived HIV risk, unjustified promotion of PrEP by clinicians, in the context of prior experiences (often

			negative) with hormonal contraceptives pills that shape doubts about personally taking PrEP.
Relationship and gender challenges	Gender roles, relationship structures, and power imbalances prevalent within some Black communities that influence HIV vulnerability	Relationship and gender dynamics	Gender roles, relationship assumptions, and power imbalances between partners (e.g. inability to negotiate protection, and ignorance of male partners' sexual behaviors) contributed to HIV vulnerability and hindered PrEP access for some Black women.
Empowerment and agency	Promoting open sexual health discussions and autonomy in health decisions for Black women	Empowerment	By promoting open and shame-free conversations, educating from a young age, framing PrEP use positively, and making PrEP readily accessible when women want it, Black women can be equipped and enabled to take control of their sexual health.
		Agency	The need for Black women to have more control, autonomy and empowerment to make informed choices about their sexual health and PrEP access by removing barriers, stigma, and judgement, and providing PrEP when women determine they want/need it rather than based on restrictive criteria. PrEP itself can provide Black women with agency over their own body in the context of infidelity concerns, inability to negotiate protection, and unawareness of male partners' sexual behaviors.
Improved HIV and PrEP knowledge	Tailored, representative, and understandable HIV & PrEP resources for Black communities.	Good HIV and PrEP knowledge	Raising HIV and PrEP knowledge amongst Black communities so they can accurately assess their own HIV risk and decide for themselves whether PrEP is a good HIV prevention option for them. This can be done via community outreach and via community-led information dissemination (ideally a combination of both). It is very important that this should be done in a culturally sensitive, accessible and representative manner.

		Culturally sensitive, accessible, representative information and education material	The need for HIV & PrEP educational resources that are tailored for and feature Black communities. This includes information that is easy to understand, uses appropriate language, represents diverse identities, and makes clear that PrEP is relevant and available to Black women.
Community engagement and advocacy	Leveraging trusted community stakeholders and peers to promote trust and PrEP uptake.	Community outreach	Educating and engaging with and within Black communities about HIV prevention and PrEP, rather than just expecting people to seek out information themselves. Outreach involves meeting people where they are, building trust, and delivering culturally relevant education and resources.
		Community-led information & education	The importance of having Black community members disseminating accurate HIV & PrEP information to improve knowledge within the community, rather than being imposed from outside. It emphasises leveraging trusted community members and community organisations to lead outreach efforts educate each other, increase awareness and fight stigma.
		Peer support & advocacy	Having peers, community members, and advocates from within Black communities providing information, encouragement, and support around accessing HIV prevention (including PrEP). This includes peers sharing their own experiences to build trust and understanding, as well as advocating for greater access, reduced stigma, and culturally appropriate services.
Addressing racialised discrimination	Addressing systemic racism in healthcare, promoting diversity, and ensuring equity.	Addressing institutional racism and representation in healthcare	Requires the recognition of institutional racism in the healthcare setting and addressing it via cultural competency from the HCPs and the SSHS, increasing diverse HCP representation across all levels, and accountability and monitoring of local and national PrEP outcomes to ensure that inequities are being addressed.

		Co-production	Actively involve and collaborate with Black communities, particularly Black women, in designing and implementing HIV and PrEP services, programs, and campaigns – rather than taking a top-down approach. This emphasises working together from the start to understand Black women's perspectives, build trust, and create interventions that genuinely meet the needs of Black women and feel safe and relevant to them.
Cultural and societal change	Normalising sexual health discussions, destigmatising HIV, and reframing PrEP perceptions	Cultural and societal change on sex, HIV & PrEP	Cultural and societal change within the Black communities, the HCP community and wider society in how we talk about sex (normalising open conversations and facing the reality that one does not wait until marriage to have sex), and HIV (destigmatising HIV as an understandable health condition rather than a moral failing) and PrEP (reframing PrEP use as a responsible self-care as part of overall health and holistic wellbeing rather than promiscuity).
Improved PrEP availability and accessibility	Changes to PrEP policies required to make PrEP more accessible to all.	Improved PrEP commissioning	The need to change how PrEP is funded, provided and delivered at a national level to increase accessibility. This includes the (1) prioritisation of sexual health and sufficient funding for existing PrEP services in the context of the broader international HIV pandemic and commitments, (2) expanding PrEP delivery beyond SSHS, (3) integrating routine PrEP conversations across healthcare setting (requires training of HCP, including those working in SSHS, so they are knowledgeable of PrEP), and (4) reducing restrictions on eligibility criteria.
		Additional PrEP commissioning in other settings	Expanding PrEP provision beyond SSHS to make PrEP more widely and easily accessible via additional commissioning in primary care, pharmacies, community settings, and online services and better integration of sexual healthcare across

			the healthcare system. This would require NHSE to allow such provision outside of SSHS.
		Reframing eligibility criteria	Revise the national PrEP guidelines away from the currently restrictive MSM-centric criteria, to promote routine discussions about PrEP and normalise it as an option for anyone at potential risk of HIV acquisition (rather than requiring evidence of prior high-risk behaviour). This would help destigmatise PrEP and its use.
