# Supplementary material for: Co-designing interventions with multiple stakeholders to address barriers and promote equitable access to HIV Pre-Exposure Prophylaxis (PrEP) in Black women in England
Source: BMC Public Health. 2025 May 17;25:1831. doi: 10.1186/s12889-025-23023-5 (PMC12085007; doi:10.1186/s12889-025-23023-5)
Supplement: Supplementary file 3 — Supplementary Material 3: Workshop co-design hand-outs provided to co-design workshop participants. This file is the detailed workshop materials provided to the study participants to guide them step by step on how to design new interventions to tackle barriers to PrEP access. [file 12889_2025_23023_MOESM3_ESM.pdf]

Step 1: What is the aim(s) of the intervention? Consider which aim is most appropriate to address the barrier or facilitator you've selected, and discuss the different options amongst yourselves.

Intervention aim	Definition	Example of intervention function
**Education**	Increasing knowledge or understanding	*Providing information to promote healthy eating*
**Convincing**	Using communication to induce positive or negative feelings or stimulate action	*Using imagery to motivate increases in physical activity*
**Incentive/motivate**	Creating an expectation of reward	*Using prize draws to induce attempts to stop smoking*
**Discourage/deter**	Creating an expectation of punishment or cost	*Raising the financial cost of alcohol to reduce excessive alcohol consumption*
**Training**	Teaching/learning imparting skills	*Advanced driver training to increase safe driving*
**Restriction**	Using rules to reduce the opportunity to engage in certain behaviour (or to increase certain behaviour by reducing the opportunity to engage in competing behaviours)	*Prohibiting sales of cigarettes to people under 18 to reduce smoking in young adults*
**Environmental restructuring/reshaping**	Changing the physical or social context	*Providing on-screen prompts for GPs to ask about smoking behaviour*
**Leading by example**	Providing an example for people to aspire to or imitate	*Using TV drama scenes involving condom use to increase safe-sex practices*
**Support**	Increasing means/reducing barriers to increase capability (beyond education and training) or opportunity (beyond environmental restructuring)	*Support for smoking cessation, medication for cognitive deficits, surgery to reduce obesity, prostheses to promote physical activity*

Step 2: What is the type of intervention? Consider which type of intervention can deliver the aim you've picked in the previous exercise, discuss the different options amongst yourselves.

Intervention type/category	Definition	Example of intervention function
**Communication/ marketing**	Using print, electronic, telephonic or broadcast media	*Conducting mass media campaigns*
**Guidelines**	Creating documents that recommend or mandate practice. This includes all changes to service provision	*Producing and disseminating treatment protocols*
**Fiscal measures (money)**	Using the tax or borrowing system to reduce or increase the financial cost	*Increasing import duties on spirits*
**Regulation (rules)**	Establishing rules or principles of behaviour or practice	*Establishing voluntary agreements on advertising*
**Legislation (laws)**	Making or changing laws	*Prohibiting sale or use*
**Environmental/ social planning**	Designing and/or controlling the physical or social environment	*Using speed bumps to reduce car speed in residential area/schools*
**Service provision (access)**	Delivering a service	*Establishing support services in workplaces, communities etc.*

<u>Help table for step 2:</u> Here's a table linking the aim of the intervention to the possible types of interventions

	Aim of intervention								
Type of intervention:	**Education**	**Convincing**	**Incentive**	**Discourage**	**Training**	**Restriction**	**Environmental restructuring**	**Leading by example**	**Support**
**Communication/ marketing**									
**Guidelines**									
**Fiscal measures (money)**									
**Regulation (rules)**									
**Legislation (laws)**									
**Environmental/ social planning**									
**Service provision (access)**									

Step 3: What is the content of the intervention? Consider the type and aim(s) of the intervention you've picked previously, and discuss the different options amongst yourselves.

- Who needs to perform the intervention? *Remember the list of all stakeholders involved in the delivery of PrEP in England*
- What must they do to implement the intervention to increase PrEP access for Black women?
- When do they need to implement the intervention?
- Where do they need to implement the intervention?
- How often do they need to implement the intervention?
- With whom do they need to implement the intervention?
