# Supplementary material for: Co-designing interventions with multiple stakeholders to address barriers and promote equitable access to HIV Pre-Exposure Prophylaxis (PrEP) in Black women in England
Source: BMC Public Health. 2025 May 17;25:1831. doi: 10.1186/s12889-025-23023-5 (PMC12085007; doi:10.1186/s12889-025-23023-5)
Supplement: Supplementary file 4 — Supplementary Material 4: Barriers voted as most important by the FG participants via a consensus-building exercise. Lists of the barriers to PrEP access that were voted as most important by the participants in each focus group stream (mixed stakeholders, HCP-only, and Black women-only), including vote counts and number of participants. [file 12889_2025_23023_MOESM4_ESM.docx]

1. **List of the modifiable barriers voted most important in the mixed stakeholder stream focus groups**

Summary of focus group 1 (01/02/2023):

- The disparity of use and *lack of trust* in health services by the black community – 4 votes out of 4 attendees;
- *Not understanding* what PrEP is or not seeing it as relevant – 4 votes out of 4 attendees;
- *Issue of access* to sexual health services – 2 votes out of 4 attendees.

Summary of focus group 2 (15/02/2023):

- PrEP *not being raised in routine health care* & sexual health consultations – 4 votes out of 5 attendees;
- Mostly marketed it as something that *gay/bisexual men need* – 3 votes out of 5 attendees;
- *Lack of awareness* of one’s own HIV "risk" acquisition – 3 votes out of 5 attendees.

Summary of focus group 3 (22/02/2023):

- *Fear/embarrassment* about going to sexual health services - 4 votes out of 5 attendees;
- *Lack of awareness*/understanding that they are eligible for PrEP/at risk of HIV - 3 votes out of 5 attendees;
- The following 3 factors came in 3rd as they received 2 votes each:
  - *Siloed services* where the focus is narrow and just on the reason for attendance e.g. contraception;
  - *Lack of representation* in PrEP promotion materials (e.g. visuals and eligibility criteria);
    - *Limited PrEP access* to Sexual Health Services.

1. **List of the modifiable barriers voted most important in the HCP-only stakeholder stream focus groups**

Summary of focus group 1 (20/02/2023):

- *Government* is not prioritising sexual health, HIV and sexual health promotion (e.g. delayed national Sexual Health strategy/action plan) – 3 votes out of 5 participants;
- Black women don't see themselves *at risk of HIV acquisition* and therefore needing PrEP (i.e. lack of knowledge) – 3 votes out of 5 participants;
- The following 3 factors came 3rd place as they received 2 votes each:
  - *Restrictive commissioning arrangements* to access PrEP exclusively via Sexual Health services;
  - The suggestion that takers of PrEP are *promiscuous*;
  - *Stigma* in general.

Summary of focus group 2 (24/02/2023):

- *Travelling long* *distances* to reach a specialist sexual health service that provides PrEP, especially for those living in rural areas & those with mobility problems – 3 votes out of 5 participants;
- *Stigma* of being labelled *promiscuous* associated with taking PrEP – 3 votes out of 5 participants;
- Previous experiences of *racial discrimination* when accessing healthcare – 3 votes out of 5 participants.

Summary of focus group 3 (07/03/2023):

- *Stigma* from the community and other healthcare professionals, therefore information given to Black women needs to be handled sensitively – 5 votes out of 6 participants;
- *Lack of awareness* of their risk of HIV acquisition – 5 votes out of 6 participants;
- *Lack of PrEP and HIV knowledge* – 4 votes out of 6 participants.

1. **List of the modifiable barriers voted most important in the Black women-only stakeholder stream focus groups**

Summary of focus group 1 (12/01/2023):

- *Lack of PrEP knowledge* – 5 votes out of 6 participants;
- *Stigma* of being “caught” the medication and being associated with HIV – 3 votes out of 6 participants;
- *Fewer sexual health clinics* locally – 3 votes out of 6 participants.

Summary of focus group 2 (26/01/2023):

- Culture of avoiding conversation about sex as it’s considered *taboo* (centred around stigma);
- *Lack of funding and commitment* from the NHS about what issues are seen as important;
- Experiences of *institutional racism*.

Summary of focus group 3 (02/03/2023):

- The information is *not being spread* to predominantly black areas – 4 votes out of 5 participants;
- *HIV stigma* and not wanting to directly engage with anything relating to it – 3 votes out of 5 participants;
- *Lack of education and awareness* about the existence of PrEP – 3 votes out of 5 participants.
