# Supplementary material for: Co-designing interventions with multiple stakeholders to address barriers and promote equitable access to HIV Pre-Exposure Prophylaxis (PrEP) in Black women in England
Source: BMC Public Health. 2025 May 17;25:1831. doi: 10.1186/s12889-025-23023-5 (PMC12085007; doi:10.1186/s12889-025-23023-5)
Supplement: Supplementary file 5 — Supplementary Material 5: Facilitators voted as most important by the FG participants via a consensus-building exercise. Lists of the facilitators to PrEP access that were voted as most important by the participants in each focus group stream (mixed stakeholders, HCP-only, and Black women-only), including vote counts and number of participants. [file 12889_2025_23023_MOESM5_ESM.docx]

1. **List of the modifiable facilitators voted most important in the mixed stakeholder stream focus groups**

Summary of focus group 1 (01/02/2023):

- *Representation* of peer advocates and community in the design, planning and delivery of PrEP advertisement and service provision – 3 votes out of 4 attendees;
- *Outreach* within ‘black’ spaces (community centres, churches, hairdressers) with information about contacting a clinic to speak more about PrEP – 2 votes out of 4 attendees;
- *Training* for a wider range of professionals to discuss and provide PrEP – 2 votes out of 4 attendees;
- The normalisation of *more mainstream access* in the settings people access healthcare – 2 votes out of 4 attendees.

Summary of focus group 2 (15/02/2023):

- *Greater awareness*of PrEP (how can they benefit from it) – 4 votes out of 5 attendees;
- *Better training for clinicians*to increase their confidence in approaching conversations about “risk” with Black women – 3 votes out of 5 attendees;
- Framing PrEP as part of *holistic health and wellbeing* – 3 votes out of 5 attendees.

Summary of focus group 3 (22/02/2023):

- *Buy-in* from services and *culturally competent* healthcare providers – 3 votes out of 5 attendees;
- *Increase awareness* by advertising campaigns (more education) – 3 votes out of 5 attendees;
- The following 4 factors came in 3^rd^ place as they received 2 votes each:
  - More *outreach;*
  - *Improve the offering* of PrEP when patients *access sexual health services* for other reasons;
  - Having healthcare providers of *similar backgrounds* (additional: speaking the same language)
  - *Increase PrEP access points* to include primary care centres and pharmacies.

1. **List of the modifiable facilitators voted most important in the HCP-only stakeholder stream focus groups**

Summary of focus group 1 (20/02/2023):

- *Better knowledge of PrEP* via media campaigns aimed at the cohort to explain and promote PrEP – 4 votes out of 5 participants;
- *Widening out access* routes to PrEP (pharmacy, online) – 3 votes out of 5 participants;
- The following 3 factors came 3rd place as they received 2 votes each:
  - *Destigmatise*the use of*sexual health measures* that have previously been used to suggest promiscuity;
  - *Reduce the stigma*of accessing Sexual Health services as a Black women;
  - Trained & trusted *community members* who are a*point of contact* for communities (community champions).

Summary of focus group 2 (24/02/2023):

- *Access to PrEP in other settings*, starting with General Practice – 4 votes out of 5 participants;
- *Seeing or hearing other Black women* talking about or using PrEP (similar to community champions) – 3 votes out of 5 participants;
- *Increased information on PrEP* readily available online that is culturally sensitive/specific and promotes the benefits/tackles stigma – 3 votes out of 5 participants.

Summary of focus group 3 (07/03/2023):

- *Wider availability/access* *in other settings* e.g. community pharmacies and GP practices like for contraceptives – 6 votes out of 7 participants;
- *More education to patients* about what makes you at risk of HIV acquisition (and therefore eligible for PrEP), AND *more education to Healthcare professionals* that Black females are at risk and should be offered PrEP – 5 votes out of 7 participants;
- *Better outreach method* to reach out to groups and communities that are at *risk of HIV acquisition* – 4 votes out of 7 participants;
- Specifically *mentioning “women”* on our website/leaflet and other *promotional material* – e.g. women may be unaware of their need, or PrEP isn't just for the MSM population – 4 votes out of 7 participants.

1. **List of the modifiable facilitators voted most important in the Black women-only stakeholder stream focus groups**

Summary of focus group 1 (12/01/2023):

- *Better knowledge* of PrEP – 4 votes out of 6 participants;
- *Wider PrEP availability* in other healthcare services (including GPs and pharmacies) – 4 votes out of 6 participants;
- Emphasis on *women empowerment* (similar to the narrative adopted for female condoms) – 2 votes out of 6 attendees.

Summary of focus group 2 (26/01/2023):

- *Hearing about PrEP* on social media and films;
- *Understanding* when one should consider taking PrEP (when they’re at risk of HIV acquisition);
- *Campaigns* that featured people who look like me (improved representation in the context of knowledge sharing).

Summary of focus group 3 (02/03/2023):

- HIV PrEP should be *part of the conversation* when going to a sexual health clinic (or during an online consultation) *for contraception or reproductive health* – 4 votes out of 5 participants;
- *More knowledge* *and promotion* throughout the NHS (including GPs & pharmacies) on PrEP and how to access it – 3 votes out of 5 participants;
- Access to *different PrEP modality* (long-acting injectable, vaginal rings…), not just the daily pills – 2 votes out of 5 participants;
- *Better advertisement* and discourse around *PrEP is for everyone* (not just for men who have sex with men) – 2 votes out of 5 participants.
