# Supplementary material for: Co-designing interventions with multiple stakeholders to address barriers and promote equitable access to HIV Pre-Exposure Prophylaxis (PrEP) in Black women in England
Source: BMC Public Health. 2025 May 17;25:1831. doi: 10.1186/s12889-025-23023-5 (PMC12085007; doi:10.1186/s12889-025-23023-5)
Supplement: Supplementary file 6 — Supplementary Material 6: Stakeholders listed as having a stake in the delivery of PrEP in England. Table categorising the stakeholders identified by each co-design stream as having a stake in PrEP delivery in England. Stakeholders are grouped into five stakeholder categories: HCPs, industry, policy-makers, patients & civil society, and researchers. Italised entries indicate stakeholders identified as currently under-used and/or needing greater involvement in PrEP delivery. [file 12889_2025_23023_MOESM6_ESM.docx]

**Stakeholders listed by workshop stream as having a stake in the delivery of PrEP in England.** Those in italics were discussed as currently being side-lined or needing to be more involved in the delivery of PrEP in England^[[1]](#footnote-1)^.

|  | Mixed stakeholders workshop | Black women-only workshop | HCP-only workshop |
| --- | --- | --- | --- |
| HCP stakeholders | - *Primary care^[[2]](#footnote-2)^* - *Pharmacies^2^* - *Online providers^2^* - Sexual health professionals (Doctors, nurses, healthcare assistants, pharmacists) - *Outreach workers (from homeless, A&E, drug and prison services)* - Public Health managers - *Student healthcare providers* | - *Sexual health professionals*, including:   - Doctors   - Nurses   - Sexual health advisors - *Online sexual health services* (offer private testing options valued by women) | - Doctors (clinical consultants, community clinicians, hospital and general practice) - Nurses (specialised nurses, HIV nurses, practice nurses and outreach nurses) - Pharmacists - Health advisors - Healthcare assistants - Social workers - Outreach teams (e.g. homeless outreach teams) - Public Health commissioners |
| Industry stakeholders | - Pharmaceutical companies - *Online provider^2^* - *Private providers* - *Social media and influencers* | - *Pharmaceutical companies* (e.g. GSK) - *Healthcare providers*, including:   - NHS   - Trusts   - Hospitals   - Pharmacies - *Any company that works in sexual and reproductive health*, including:   - Durrex   - Boots - *Any company that works in the personal hygiene and pleasure sector* (e.g. Ann Summers, Love honey and Veet) | - Pharmaceutical companies (e.g. Gilead who make Descovy and Truvada and other who make generics) - Policy advocates - Employers with a large workforce and their occupational health departments - Black African and Women's advocacy organisations - Formulary departments |
| Policy-makers stakeholders | - National government (Westminster) - Local Authorities (LA) - NHS - UK Health Security Agency (UKHSA) - NHS Integrated Care Boards (ICBs) - UK Professional associations:   - British Association for Sexual Health and HIV (BASHH)   - British HIV Association (BHIVA)   - *Faculty of Sexual and Reproductive Healthcare (FSRH)* - *Educational institutions* | - *National government* (including Chancellor who needs to give more funding to the sector) - *NIHR* - *NICE* - *Educational policy* (participants were not sure who that stakeholder was) | - Politicians and All-party parliamentary group - NICE - NHS - Local authorities, including the English HIV and Sexual Health Commissioners’ Group - BASHH - BHIVA - Professional regulatory bodies (General Medical Council, General Pharmaceutical Council, Nursing and Midwifery Council) - Medicines and Healthcare products Regulatory Agency (MHRA) - British Medical Association (BMA) |
| Patients & civil society stakeholders | - *Outreach workers^[[3]](#footnote-3)^* (from Non Governmental Organisations & charities) - Patients - Social workers - *Educational bodies* - *Peer mentors from the Black community* - Community groups - *Religious settings* - *Events/festivals* | - *Schools* (and their sex ed curriculum) - Black women - Healthwatches - Community representatives & champions - *Charities and other grassroot organisations*, including:   - Black and women empowerment groups   - Human rights groups   - Mental health groups (example: Mind)   - Social and health innovation groups   - Cancer groups   - Social housing associations for women (example: Youth Aid)   - Is there a charity set up to target Black people with PrEP information? | - Black women, including community champions - HIV charity sector organisations (Terrence Higgins Trust, NAM, National AIDS Trust, Positively UK, NAZ project, local and regional as well as national organisations) - Community based organisations representing minority groups - Anyone who is sexually active - Faith-based stakeholders |
| Researchers stakeholders | - *Educational bodies* - *Think tanks* - *Funders that can require inclusive research:*   - National institute for Health and Care research (NIHR)   - Medical Research Council (MCR)   - UK Research and Innovation (UKRI) | - Universities - Clinical trial team | - BASHH - NIHR ARC - Funders that can support inclusive and equitable research (NIHR, NICE, Medical Research Council, UK Research and Innovation) - PPIE and peer researchers - NHS Trusts (the biggest one with HIV prevention, Chelsea and Westminster trust) - Pharmaceutical companies - Arts organisations - HIV charities and organisations |

1. HCP-only workshop does not have any stakeholder listed in italics due to the technical issues of Zoom that did not save those that were considered as being sidelined. [↑](#footnote-ref-1)
2. As PrEP is currently only available in SSHS, provision of PrEP in the primary care setting, pharmacies and online providers would represent a structural change in access. Until then, these HCP could be at the forefront of *raising awareness and understanding of PrEP* by making use of the [Making Every Contact Count approach](https://www.hee.nhs.uk/our-work/population-health/making-every-contact-count-mecc). [↑](#footnote-ref-2)
3. Outreach workers are listed twice, as they can work within the healthcare system, for homeless, A&E, drug and prison services, and within the charity sector. [↑](#footnote-ref-3)
